# Identification of an Unsaturated Hydroxy Fatty Acid Antitumor Oxylipin from Brown Alga *Scytosiphon lomentaria*: (*E*)-6-Hydroxyoctadec-4-enoic Acid (NP-011548)

**DOI:** 10.3390/md24100363

**Published:** 2026-10-19

**Authors:** Matteo Mauceri, Abdul-Salam Juhmani, Roberta Zangrando, Michele Gallo, Flavio Rizzolio, Adriano Sfriso, Sarah Pizzini, Marco Roman, Andrea Gambaro, Carlotta Granchi, Tiziano Tuccinardi, Giuseppe Forlani, Andrea Augusto Sfriso

**Affiliations:** 1 Department of Environmental Sciences, Informatics and Statistics, Ca’ Foscari University of Venice, Via Torino, 155, 30172 Venezia, Italy; matteo.mauceri@unive.it (M.M.); sarah.pizzini@cnr.it (S.P.); marco.roman@unive.it (M.R.); gambaro@unive.it (A.G.); 2 Department of Biology and Biotechnology, Faculty of Science, The Hashemite University, Zarqa 13133, Jordan; a.juhmani@hu.edu.jo; 3 National Research Council, Institute of Polar Sciences (CNR-ISP), Via Torino, 155, 30172 Venezia, Italy; 4 Department of Molecular Sciences and Nanosystems, Ca’ Foscari University of Venice, Via Torino, 155, 30172 Venezia, Italy; gallomke@unive.it (M.G.);; 5 Pathology Unit, Centro di Riferimento Oncologico di Aviano (CRO), Via F. Gallini, 2, 33081 Aviano, Italy; 6 National Research Council, Institute for Marine Biological Resources and Biotechnology (CNR-IRBIM), Largo Fiera della Pesca, 2, 60125 Ancona, Italy; 7 Fano Marine Center, The Inter-Institute Center for Research on Marine Biodiversity, Resources and Biotechnologies, Viale Adriatico, 1/N, 61032 Fano, Italy; 8 Department of Pharmacy, University of Pisa, Via B. Pisano, 6, 56126 Pisa, Italy; carlotta.granchi@unipi.it (C.G.); tiziano.tuccinardi@unipi.it (T.T.); 9 Department of Life Sciences and Biotechnology, University of Ferrara, Via L. Borsari, 46, 44121 Ferrara, Italy; flg@unife.it

**Keywords:** oxylipin, anticancer, ROS, selective, extract, algae, *Scytosiphon lomentaria*

## Abstract

Marine brown algae are an important source of bioactive metabolites with promising anticancer potential. *Scytosiphon lomentaria* has repeatedly shown selective antitumor activity, although the active metabolite associated with its lipophilic fractions had not previously been identified. Here, five solvent extracts were screened and a bioassay-guided primary and secondary fractionation approach, using the less-active related species *Scytosiphon dotyi* as a comparator, was combined with high-resolution mass spectrometry to identify the oxylipin (*E*)-6-hydroxyoctadec-4-enoic acid (NP-011548) as a metabolite endowed with significant antitumor activity. The pure compound selectively reduced the viability of human breast (MDA-MB-231) and ovarian (Kuramochi) cancer cells while exhibiting limited toxicity towards normal fibroblasts. Comparative testing with the structural analogs 6-hydroxystearic acid (6-HSA) and 6-oxostearic acid (6-OSA) further indicated that the C4 double bond and the C6 hydroxy group may contribute to the selective activity of NP-011548. NP-011548 induced delayed reactive oxygen species accumulation without activating caspase-3/7 and caused extensive mitochondrial degeneration, lysosomal accumulation, chromatin condensation and plasma membrane alterations, supporting the involvement of a caspase-independent cell death. These findings identify NP-011548 as a candidate metabolite underlying the antitumor activity of *S. lomentaria*, expanding the biological relevance of marine oxylipins and supporting their potential as a source of novel anticancer agents.

## 1. Introduction

Over the past few decades, marine organisms have attracted considerable attention in biomedical research for their ability to produce a wide range of secondary metabolites with potential pharmaceutical applications [1,2,3]. Among marine macroalgae, the brown algae (Phaeophyta, comprising an estimated 1500–2000 species) represent one of the most chemically diverse groups, standing out due to the presence of bioactive compounds such as terpenes, polyphenols, acetogenins, sulfated polysaccharides, carotenoids, and mixed-biosynthesis metabolites [1,2,4,5,6]. Phaeophyta are almost exclusively marine, ranging from microscopic filamentous forms to the macroscopic kelps, and they often dominate temperate environments.

A significant portion of the secondary metabolites derived from brown algae arises through terpenoid biosynthesis and many of these compounds display noteworthy biological activities, including ecological functions as pheromones involved in reproductive processes or as chemical defenses against fouling, predation, and competition [7]. From a biomedical perspective, numerous marine derived compounds, particularly those found in brown algae, have demonstrated promising activities such as antiviral, antimicrobial, and anticancer effects [6,8].

The discovery of novel anticancer compounds from marine organisms has become of particular interest due to the structural diversity and potential therapeutic properties of marine natural products. Although extensive investigations have focused on well-known brown algal genera such as *Laminaria*, *Fucus*, *Dictyota*, *Sargassum*, and *Cystoseira*, many other genera remain poorly explored and may represent a rich source of untapped bioactive compounds with significant therapeutic potential [5].

In this context, the genus *Scytosiphon* (order Scytosiphonales) represents a promising but relatively underexplored source of bioactive metabolites with potential pharmaceutical applications including anticancer agents. Although it is less studied compared to some other brown algae, the literature evidence suggests that *Scytosiphon* species may produce unique metabolites capable of exerting antimicrobial [9], anti-inflammatory, and antioxidant [10] properties and growth-inhibitory effects on cancer cells [11]. As in many other brown algae, several bioactive compounds have been reported in the widespread species *Scytosiphon lomentaria* (Lyngbye) Link, including: phlorotannins [12], fucoxanthin [13,14,15], and sulfated polysaccharides such as fucoidan [16,17,18]. Notably, isoprenoids, the most common bioactive compounds in Phaeophyta, have never been documented in the order Scytosiphonales [5]. Early screening studies demonstrated that *S. lomentaria* powder fed to mice exhibited a pronounced antitumor activity [11], outperforming several other brown algae species. These findings were subsequently supported by studies reporting selective cytotoxic activity in ethanolic and ethyl acetate *S. lomentaria* extracts [19,20]. However, despite the consistent demonstration of antitumor activity, the metabolites responsible for these effects had not been identified. Given that many bioactive constituents, such as phlorotannins and fucoxanthin, are shared with other brown algae but do not exhibit comparable antitumor potency [11], it is plausible that *S. lomentaria* synthesizes unique or previously uncharacterized metabolites responsible for its selective cytotoxic activity. Therefore, *S. lomentaria* represents an attractive candidate for discovery and characterization of selective marine-derived antitumor metabolites.

The objectives of this study were to (i) identify the metabolite most strongly associated with the selective antitumor activity of *S. lomentaria* using bioassay-guided fractionation and high-resolution mass spectrometry (HRMS); (ii) validate the biological activity of the identified compound using purified standards and structural analogs; and (iii) obtain preliminary mechanistic insights into its mode of action through functional and ultrastructural characterization. More broadly, this study addresses the still unresolved chemical basis of the selective antitumor activity of *S. lomentaria* and extends current knowledge of marine oxylipins by combining the identification of a naturally occurring algal oxylipin with comparative structure–activity analysis using closely related oxygenated fatty-acid (OFA) analogs. This approach allows the biological relevance of specific structural features of the marine oxylipin to be explored in relation to cytotoxicity and selectivity.

## 2. Results

### 2.1. Bioactive Compound Separation

The chloroform extract from brown alga *S. lomentaria* exhibited promising selective anticancer activity, as evidenced by cytotoxicity assays showing significant inhibitory effects on the viability of the tumor cell lines Kuramochi (ovarian cancer, IC_50_: 73.6 ± 2.3 µg mL^−1^) and MDA-MB-231 (breast cancer, IC_50_: 78.6 ± 10.7 µg mL^−1^), while sparing the normal human fibroblast cell line MRC-5 (IC_50_ > 300 µg mL^−1^). The extract from the related species *Scytosiphon dotyi* M.J. Wynne, used as a term of comparison, showed much lower activity. To identify the fractions responsible for this selective activity, the chloroform extract underwent primary fractionation using gel permeation chromatography (GPC), leading to four fractions (F1–F4), which were collected based on distinct retention times (RTs). Among these, fraction F2 displayed potent cytotoxic activity, closely mirroring the selectivity profile observed in the original chloroform extract. Specifically, F2 showed cytotoxicity (Table 1) toward the MDA-MB-231 cell line (IC_50_: 25.4 ± 4.6 µg mL^−1^), moderate activity against the Kuramochi cell line (IC_50_: 141 ± 13 µg mL^−1^), and very low activity against normal human fibroblast cell line MRC-5 (IC_50_ > 300 µg mL^−1^). Given these results, fraction F2 was selected for secondary fractionation by reversed-phase solid phase extraction (SPE), resulting in seven subfractions (F2A–F2G). Among these subfractions, F2E exhibited the most promising anticancer profile, maintaining strong selective activity against the MDA-MB-231 cells (IC_50_: 30.6 ± 5.3 µg mL^−1^) and moderate effects on the Kuramochi cells (IC_50_: 116 ± 24 µg mL^−1^). Therefore, subfraction F2E was chosen for further chemical characterization through high-performance liquid chromatography (HPLC) coupled with HRMS.

### 2.2. HPLC-HRMS Investigation for the Identification of Bioactive Compounds

The potential antitumor activity of the F2E fraction of *S. lomentaria* was preliminarily tested by comparing its effect with that of *S. dotyi*, which had been shown to be inactive (Table 1). In order to evaluate which molecular species could be present in the F2E fraction of *S. lomentaria* that made it active against tumor cells, the following samples were analyzed (three replicates) by HPLC-HRMS: *S. lomentaria* active F2E fraction (F2E-SL), *S. dotyi* inactive F2E fraction (F2E-SD), extracts from MDA-MB-231 breast tumor cells treated with the active fraction F2E-SL obtained 1 h and 48 h after the treatment, extract from untreated MDA cells, and DMEM media supplemented or not with active fraction F2E-SL. Data analysis was performed using the Compound Discoverer^TM^ 3.3 software (Figure S1). The species identified by the software were filtered for a Δmass (mass error between the experimental exact mass and calculated formula) of 3 ppm, the ratio of areas of the same ionic species in the sample and in the control > 3, and a *p*-value lower than 0.05 (Table 2 and Figure S2). The ratios were used to identify the compound most present in *S. lomentaria*, which was accumulated in the cells and was present mainly in the extract and not in the DMEM medium.

The analysis identified many metabolites in both positive and negative ionization modes, but none was common for all the experiments, except for the negative ionic species [M − H^−^]^−^ *m*/*z* 297.2428, corresponding to the neutral formula C_18_H_34_O_3_.

The software identified the molecule as the endogenous metabolite NP-011548 in the mzCloud™ library (mzCloud™ ID: Autoprocessed-6496), based on the comparison of mass spectra fragmentation acquired from the raw file and the mzCloud™ reference (Figure S3), with a Best Match score of 98.9. The mzCloud™ mass spectral library is built from commercially available reference standards, each of which is individually analyzed using Thermo Scientific™ Orbitrap™ Hybrid and Orbitrap Tribrid™ mass spectrometers. The high resolving power and mass accuracy of these analytical platforms provide a high degree of confidence in the resulting MS, MS/MS, and multi-stage MS^n^ data.

The compound NP-011548 “(*E*)-6-hydroxyoctadec-4-enoic acid” is classified as a hydroxy fatty acid and features a hydroxy functional group at C6 and a double bond at C4. The structure was confirmed at level of confidence L1 [21] using an Orbitrap Exploris^TM^ 120 comparing *m*/*z* 297.2428 in the F2E-SL algae fraction with the analytical standard NP-011548. The exact mass, fragmentation spectrum, and RT (32.21 min) for the unknown compound *m*/*z* 297.2428 in the algae and the NP-011548 analytical standard match (Figure S4).

### 2.3. Cytotoxicity, ROS, and Caspase Activation

In order to understand the structural factors influencing anticancer selectivity, NP-011548 pure compound was tested comparatively with two structural analogs: 6-hydroxystearic acid (6-HSA), which lacks the double bond and contains a hydroxy group at position 6; 6-oxostearic acid (6-OSA), characterized by a keto group at C6 and also lacking a double bond. Cytotoxicity assays revealed substantial differences in their activity profiles (Table 3). NP-011548 demonstrated moderate cytotoxicity against Kuramochi (IC_50_: 91.9 ± 7.0 µM) and MDA-MB-231 (IC_50_: 91.5 ± 7.1 µM) cell lines, with minimal cytotoxic effects on normal MRC-5 cells (IC_50_ >200 µM), indicating moderate selectivity. In contrast, 6-HSA exhibited notably stronger cytotoxicity against all tested cell lines, including the normal cell line, displaying very low IC_50_ values (MDA-MB-231: 3.34 ± 0.45 µM; Kuramochi: 18.7 ± 1.2 µM; MRC-5 proliferation: 12.1 ± 1.6 µM), thus indicating lower selectivity and higher general toxicity. The 6-OSA showed intermediate cytotoxicity, with potent effects against MDA-MB-231 (IC_50_: 17.2 ± 3.5 µM), but moderate activity against Kuramochi (IC_50_: 109 ± 5 µM) and MRC-5 cells (IC_50_ proliferation: 79.0 ± 7.7 µM), suggesting partial selectivity compared to NP-011548. These observations indicate that structural features such as the C4 double bond and the hydroxy group at C6 may contribute more to cellular selectivity than to absolute cytotoxic potency.

**Figure 1:**
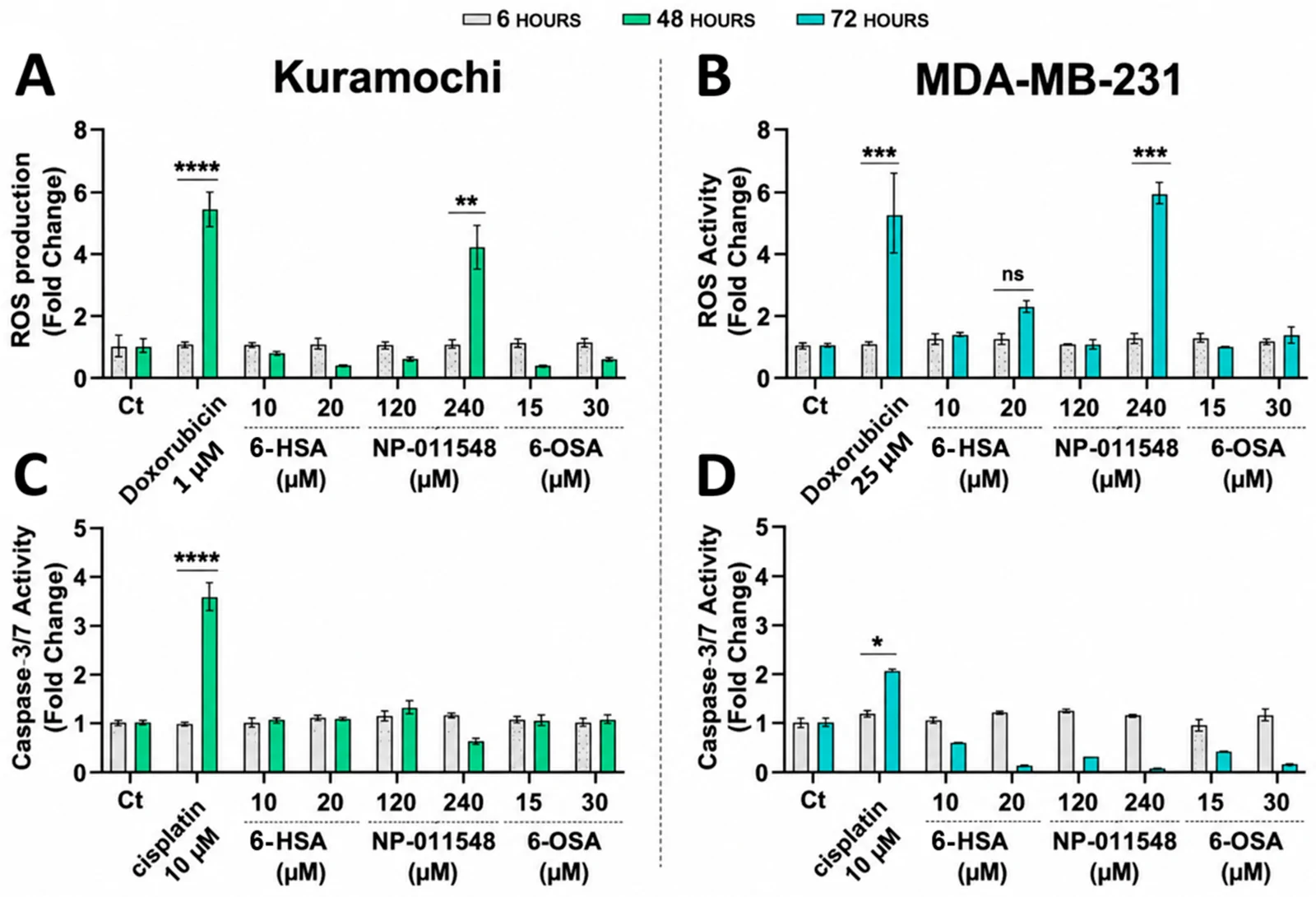
ROS production after the treatment with Doxorubicin 1 µM, 6-HSA (10 µM, 20 µM), NP-011548 (120 µM, 240 µM), and 6-OSA (15 µM, 30 µM). The treatment time was for (**A**) Kuramochi at 6 and 48 h and for (**B**) MDA-MB-231 at 6 and 72 h. Caspase-3/7 activity after the treatment with cisplatin 10 µM, 6-HSA (10 µM, 20 µM), NP-011548 (120 µM, 240 µM), and 6-OSA (15 µM, 30 µM). The treatment time was for (**C**) Kuramochi at 6 and 48 h and for (**D**) MDA-MB-231 at 6 and 72 h. Two-tailed *t*-test: * *p* < 0.05; ** *p* < 0.01; *** *p* < 0.001; **** *p* < 0.0001; ns: not significant.

The ROS production (Figure 1A,B) was evaluated in Kuramochi and MDA-MB-231 cell lines and compared with 6-HSA and 6-OSA. Generation of ROS in neither cell line was significantly induced by 6-HSA and 6-OSA, whereas NP-011548 significantly stimulated ROS generation in both cell lines after 48 h (Kuramochi) and 72 h (MDA-MB-231).

The caspase-3/7 activation was evaluated (Figure 1C,D); however, none of the tested compounds induced significant caspase-3/7 activity under the experimental conditions.

These results indicate that the structural differences, particularly the presence and position of the double bond and the nature of the functional group at C6, play crucial roles in determining both cytotoxic potency and cellular selectivity.

### 2.4. Ultrastructural Alterations

**Figure 2:**
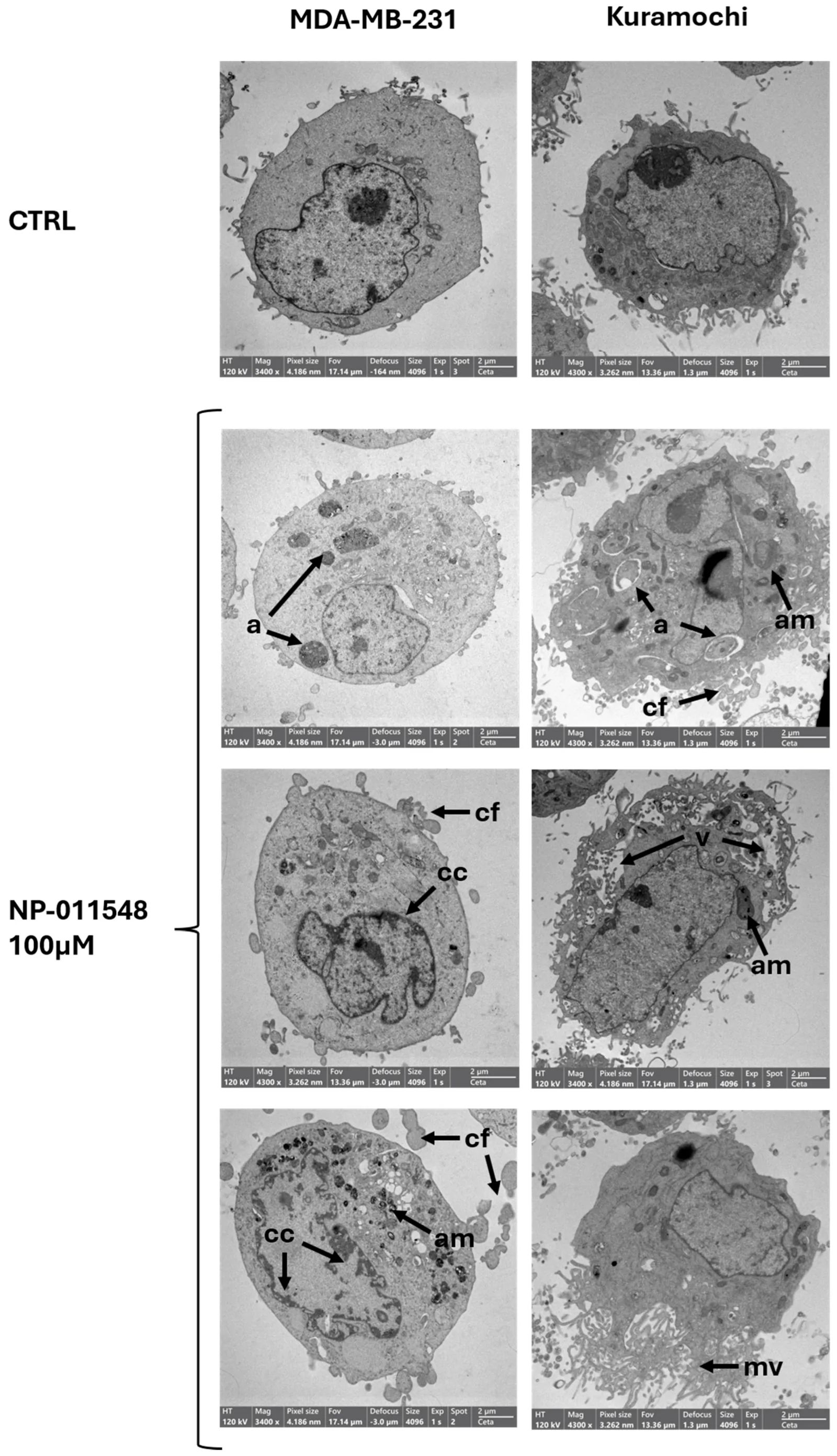
Transmission electron microscope images of MDA-MB-231 and Kuramochi cancer cell lines treated for 24 h with NP-011548 in comparison with control untreated cells (CTRL). Legend—a: autophagic structure; am: aberrant mitochondria; cc: chromatin condensation; cf: cytoplasmic fragments; mv: microvilli; v: vacuolization.

Transmission electron microscopy revealed extensive and distinct ultrastructural alterations following NP-011548 treatment in both MDA-MB-231 breast cancer cells and Kuramochi ovarian cancer cells (Figure 2). In untreated controls, both cell types exhibited a continuous and well-defined plasma membrane, with an organized cytoplasm containing structurally intact organelles. Mitochondria retained clear cristae and a dense, uniform matrix, while nuclei displayed evenly dispersed chromatin and prominent nucleoli—hallmarks of metabolically active and viable cells. Cytoplasmic vacuolization was minimal or absent.

Upon drug exposure, both cell types displayed pronounced morphological alterations, although differences emerged in the extent and nature of the changes.

At the level of the plasma membrane, MDA-MB-231 cells showed irregular contours and membrane blebbing. Kuramochi cells, however, exhibited more severe alterations, including extensive blebbing, pronounced protrusions, and local disruptions of membrane continuity. Cytoplasmic fragmentation was observed, particularly in Kuramochi cells.

Cytoplasmic vacuolization was moderate in NP-011548-treated MDA-MB-231 cells, consistent with the activation of autophagy, lysosomal response, and endoplasmic reticulum (ER) stress. Kuramochi cells, in contrast, exhibited widespread and massive vacuolization, suggesting more severe cellular stress and widespread cytoplasmic degradation.

Mitochondria in treated MDA-MB-231 cells appeared swollen, with disrupted or absent cristae, indicating mitochondrial dysfunction and a loss of energetic capacity. Kuramochi cells displayed even more dramatic damage: mitochondria were highly swollen, fragmented, and showed complete loss of internal structure, pointing to a collapse of mitochondrial integrity.

Nuclear alterations were also prominent, especially in MDA-MB-231, chromatin condensation, increased peripheral heterochromatin, and nuclear envelope invaginations were observed.

An increased number and size of electron-dense bodies, lysosomes containing degraded mitochondria, as well as double-membraned autophagic vesicles, were observed in MDA-MB-231 and Kuramochi cells after treatment. This supports a strong activation of lysosomal and catabolic pathways as part of the cellular stress response and autophagy.

Furthermore, ER dilation and disorganization were evident in both cell lines, highlighting ER stress as a contributing factor to drug-induced damage.

Overall, while untreated cells retained a well-organized, viable ultrastructure, drug-treated cells exhibited profound morphological disruption. The pattern of cellular damage differed between the two cell lines, with Kuramochi cells displaying more severe morphological alterations despite similar IC_50_ values. These findings suggest that drug exposure triggers a multifaceted cytotoxic response with the Kuramochi ovarian cancer cells exhibiting higher susceptibility and more extensive structural deterioration compared to the MDA-MB-231 breast cancer cells.

## 3. Discussion

Marine brown algae have long been recognized as a rich source of structurally diverse bioactive metabolites with considerable pharmaceutical potential, including compounds displaying selective anticancer activity. Among these, *S. lomentaria* attracted attention more than three decades ago after crude organic extracts and seaweed powder were shown to inhibit tumor growth both in vivo and in vitro (Table S1). Several bioactive compounds and fractions have been identified in *S. lomentaria*, suggesting that its anticancer activity resulted from multiple synergistic components rather than a single active molecule. However, despite few independent studies confirming its cytotoxic potential, the bioactive molecule(s) responsible for this activity within the lipophilic fraction remained unidentified. In the present study, a bioassay-guided fractionation approach combined with HRMS enabled the identification of (*E*)-6-hydroxyoctadec-4-enoic acid (NP-011548) as the major compound associated with the selective antitumor activity of the chloroform extracts and derivative fractions of *S. lomentaria*. Notably, chloroform and ethyl acetate solvents possess very similar polarity indices (4.1 and 4.4, respectively); therefore, the bioactive ethyl acetate fraction previously reported by Kim et al. (2004) [20] is likely to contain metabolites comparable to those isolated in the present study, supporting the hypothesis that NP-011548 may account, at least in part, for the cytotoxic activity described in earlier investigations. Interestingly, antitumor activity has never been reported for the closely related species *S. dotyi*. Consistent with these observations, none of the *S. dotyi* extracts or fractions tested in the present work showed significant cytotoxic effects. This difference proved particularly valuable during the metabolomic analysis, as *S. dotyi* provided an appropriate phylogenetically related but biologically inactive comparator, allowing us to hypothetically exclude metabolites shared between the two similar species and substantially narrow the search for compounds specifically associated with the antitumor activity of *S. lomentaria*.

NP-011548 belongs to the oxylipin family, a diverse class of OFA derivatives increasingly recognized as signaling molecules involved in stress responses, defense mechanisms, and grazing deterrence [22,23]. These compounds originate from the oxidative metabolism of polyunsaturated fatty acids (PUFAs), in which primary lipid hydroperoxides generated during lipid peroxidation are subsequently converted into a wide range of oxygenated metabolites, including hydroxy-, epoxy-, dihydroxy-, trihydroxy-, and keto-fatty acids; collectively known as oxylipins [24]. As reported by Pertami et al. (2024) [25], NP-011548 belongs to a family of linoleic acid-derived oxylipins together with 9-hydroperoxy-octadecadienoic acid (9-HpODE), (15*Z*)-9,12,13-trihydroxy-15-octadecenoic acid, and 13(*S*)-hydroperoxylinolenic acid (13(*S*)-HPOTrE). Although NP-011548 has been associated with a family of C18-derived oxylipins, oxylipin biosynthesis in marine organisms is not restricted to C18 fatty acid precursors and may vary substantially among algal taxa. For example, Orefice et al. (2022) [26] showed that in diatoms, oxylipins are predominantly derived from C20 PUFAs, particularly eicosapentaenoic acid (EPA), and, in some species, from docosahexaenoic acid (DHA). This highlights the taxon-dependent diversity of oxylipin biosynthetic pathways in marine algae.

Several marine algal oxylipins from many different algal taxa [23,26,27] have previously been reported to exert anticancer activity through distinct molecular mechanisms. NP-011548 has previously been identified in shield aralia *Polyscias scutellaria* (Burm.f.) Fosberg flavored Greek yogurt following fermentation [25] and in ‘Hass’ avocado peel hexane extract associated with antibacterial activity [28], but no evidence of anticancer activity has ever been reported. Therefore, its potential role can only be inferred from structurally related OFAs. In this regard, both saturated oxo fatty acids (SOFAs) and saturated hydroxy fatty acids (SHFAs) have recently emerged as endogenous bioactive lipids exhibiting selective antiproliferative activity against several human cancer cell lines [29,30]. Among the SOFAs, 6-OSA and 7-oxostearic acid (7-OSA) were identified as the most potent growth inhibitors against A549 lung carcinoma cells, while displaying minimal effects on non-tumorigenic endothelial cells among different isomers [29]. Their antiproliferative activity was associated with suppression of the STAT3/c-Myc signaling axis, a major regulator of cancer cell proliferation and survival. Similarly, the structurally related hydroxy isomer 7-(*R*)-hydroxystearic acid (7-HSA) exhibited potent growth inhibitory activity against lung, colon, and astrocytoma cell lines [30]. Although 6-HSA (the most structurally similar compound to NP-011548) was not included in the structure–-activity relationship study of Kokotou et al. (2020) [30], the marked activity of the corresponding 6-OSA suggests that oxygenation at the C6 position may represent a favorable structural feature for biological activity [29]. The present study was not designed to assess enantioselective structure–activity relationships. The stereochemistry of the NP-011548 standard was not defined by the supplier, while 6-HSA was commercially listed under the designation “6-DL-hydroxystearic acid”. Therefore, the observed biological effects refer to the tested materials as supplied and should be interpreted in terms of broad structural differences rather than specific enantiomeric effects. Among SHFAs, 7-HSA inhibited cancer cell growth through STAT3 downregulation and cell-cycle arrest, without inducing caspase-3 activation. Similarly, NP-011548 in our data did not activate caspase-3/7 despite significantly reducing tumor cell viability, indicating that canonical apoptosis is unlikely to represent the predominant mechanism of cell death. Instead, prolonged exposure to the compound (up to 72 h) resulted in delayed ROS accumulation and was associated with extensive mitochondrial disruption, massive cytoplasmic vacuolization, accumulation of autophagic vesicles and lysosomes containing degraded mitochondria, ER dilation, and profound plasma membrane alterations. Together, these observations provide preliminary mechanistic evidence consistent with a caspase-independent mode of cell death driven by progressive oxidative and organellar stress rather than the rapid execution program typical of intrinsic apoptosis. Among the currently described forms of regulated cell death, the phenotype observed in the present study shares several morphological features with autoschizis [31]. Originally described in response to severe oxidative stress, autoschizis is characterized by progressive cytoplasmic self-excision, extensive cytoplasmic vacuolization, mitochondrial degeneration, accumulation of lysosomal structures, plasma membrane distortion, and limited or absent activation of executioner caspases. From a therapeutic perspective, the ability to trigger a caspase-independent mode of cell death may represent an attractive feature. Resistance to apoptosis is a hallmark of tumor progression and a major determinant of resistance to conventional anticancer therapies, which largely rely on activation of the apoptotic machinery. Consequently, alternative forms of regulated cell death have been proposed as potential therapeutic strategies for overcoming apoptosis resistant tumors [32,33].

Overall, this work identifies NP-011548 as a bioactive oxylipin metabolite underlying the selective anticancer activity of *S. lomentaria*, significantly expanding the known pharmacological profile of this marine species. Beyond its selective cytotoxicity, our findings reveal a mechanism distinct from classical apoptosis and provide evidence supporting the activation of a caspase independent cell death program. These results open new perspectives for exploiting marine oxylipins as leads for the development of therapeutic strategies targeting apoptosis resistant tumors.

## 4. Materials and Methods

### 4.1. Sample Collection, Preparation, and Extraction

*S. lomentaria* and *S. dotyi* are brown macroalgae characterized by tubular, filamentous thalli reaching 30–50 cm in length. Macroscopically, the two species can be distinguished by thallus diameter and the presence of constrictions along the thallus. In addition, *S. lomentaria* is characterized by the presence of mucilaginous cells (ascocysts) among the cortical cells, whereas these structures are absent in *S. dotyi*. Fresh thalli were manually collected between February and March from the Venice Lagoon (Italy) in proximity of Pellestrina Island on the artificial banks and on the basement of fishermen fishing huts. The samples were taxonomically identified [34], freeze-dried, and ground to fine powder using a mincer. Identification sheets including photographs of the two species investigated are provided in the Supplementary Material (Figure S5—*S. lomentaria*—and Figure S6—*S. dotyi*–). The algae powder was then extracted in two recoveries (30 mL + 15 mL for each solvent) using solvents of different polarity: Milli-Q water, methanol, acetone, chloroform, and hexane. All samples underwent cold ultrasound extraction for 30 min. The aqueous extract was processed with two freeze–-thaw cycles to help extraction. The samples were then centrifuged at 4500 rpm for 15 min, and the supernatants were transferred to new tubes. The solvent was evaporated under a fume hood at room temperature for 48–72 h. The residues were transferred to pre-weighed glass vials, and any remaining solvent traces were removed under nitrogen flow and further lyophilized overnight under high vacuum. The final weight of each extract was recorded. The aqueous extract was frozen and subsequently lyophilized. The overall extraction and subsequent fractionation workflow is summarized in the Supplementary Material (Figure S7).

### 4.2. Fractionation of the Extracts

The chloroform extracts of *S. lomentaria* and *S. dotyi* were subjected to size-exclusion fractionation by GPC using a column packed with spherical neutral beads of porous styrene-divinylbenzene copolymer and preconditioned with a cyclohexane:ethyl acetate mixture (1:1; *v*/*v*). The algal extract (dissolved in cyclohexane:ethyl acetate) was then filtered through a PTFE membrane and loaded onto the GPC system in two successive 1 mL injections. Elution (1 mL min^−1^) yielded four fractions (F1—10 to 15 min; F2—15 to 20 min; F3—20 to 35 min; F4—35 to 45 min) collected by volume in clean vials. Each fraction was concentrated by gentle nitrogen flow at 23 °C to approximately 100 μL, then brought up to 1 mL with methanol.

Fraction F2 (initially 100 μL) was diluted to 20 mL total volume with ultrapure water, then loaded onto a conditioned C18 SPE cartridge. Sequential elution with increasing methanol proportions in ultrapure water (from 0:100% to 100:0%) yielded 6 subfractions (F2A-…-F2F), with an additional chloroform wash (F2G). All subfractions were re-concentrated by nitrogen flow, transferred to new vials, and frozen. Finally, they were lyophilized for 24 h to remove any residual solvent and water.

### 4.3. Drug Synthesis and Purchase

**Figure 3:**
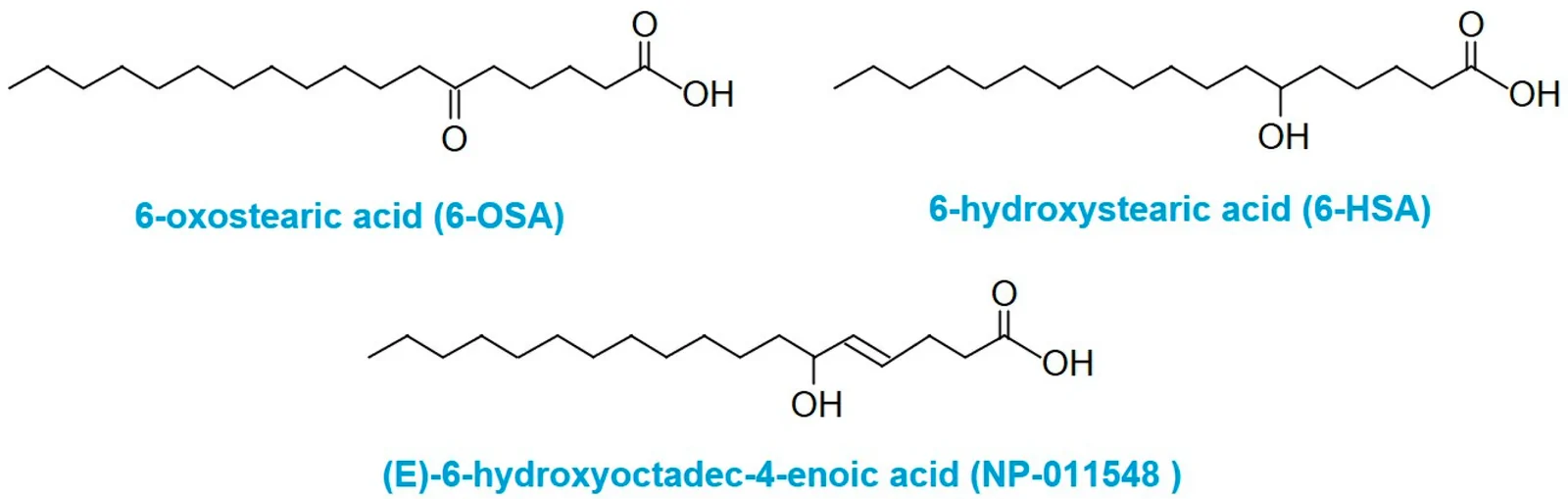
Structural comparison of NP-011548 with the saturated hydroxy fatty acid (6-HSA) and the saturated oxo fatty acid (6-OSA) used in this study.

The OFAs used both for in vitro tests and to instrumentally confirm the identity of the unknown F2E-SL active compound were both commercially purchased and synthesized. The standard NP-011548 was purchased from Mcule, Inc., Palo Alto, CA, USA (019-937-247) and according to the supplier information, NP-011548 contains one stereogenic center, but no absolute R/S configuration assigned. The OFA structural analogs 6-HSA and 6-OSA were purchased from Larodan Research Grade Lipids (Solna, Sweden) through its Italian distributor, Spectra 2000 S.r.l. (Rome, Italy) and synthesized as in Batsika et al. (2021) [29], respectively (Figure 3).

### 4.4. Extracts and Drugs Cytotoxicity

The half maximal inhibitory concentrations (IC_50_) of *S. lomentaria* and *S. dotyi* extracts and fractions, as well as of the OFA compounds 6-HSA, 6-OSA, and NP-011548, were determined in Kuramochi ovarian cancer cells, MDA-MB-231 breast cancer cells, and non-tumoral MRC-5 fibroblasts. Compounds and dried extracts were dissolved in DMSO. Cells were seeded in 96-well plates at a density of 1500 cells per well for Kuramochi and MDA-MB-231 cells and 8000 cells per well for MRC-5 cells. Kuramochi cells were maintained in RPMI-1640 medium supplemented with 10% FBS and 1% penicillin–streptomycin; MDA-MB-231 cells in DMEM supplemented with 10% FBS and 1% penicillin–streptomycin; and MRC-5 cells in MEM supplemented with 10% FBS, 1% penicillin–streptomycin, 1% non essential amino acids, 1% [sodium pyruvate], and 1% sodium bicarbonate. Cells were incubated at 37 °C in a humidified atmosphere containing 5% CO_2_. On the following day, cells were treated with six successive tenfold dilutions of the methanol, acetone, chloroform, hexane, and aqueous extracts, starting from 1 mg mL^−1^, as well as with the corresponding primary (F1–F4) and secondary (F2A–F2G) GPC/SPE fractions. Each concentration was tested in triplicate, and cisplatin was used as a positive control. After 96 h of treatment, cell viability was assessed using the CellTiter-Glo^®^ Luminescent Cell Viability Assay on a Synergy H1 plate reader. IC_50_ values were calculated by nonlinear regression using the GraphPad Prism (Version 9.5.1). Treatment concentrations were selected on the basis of preliminary dose–response experiments and adapted to the sensitivity of each cell line and the specific experimental endpoint.

### 4.5. Cellular Accumulation of Extract Fractions

MDA-MB-231 breast cancer cells were seeded in 6-well plates at a concentration of 1 × 10^6^ cells per plate. The following day, cells were treated with *S. lomentaria* F2E fraction (F2E-SL) and *S. dotyi* F2E fraction (F2E-SD) for 1 and 48 h. The following controls were included: DMEM medium alone (blank control) and DMEM medium containing F2E-SL or F2E-SD (positive control). At the end of each treatment period, cells were detached using 0.25% trypsin–EDTA, collected by centrifugation, washed with PBS to remove residual extracellular compounds, and extracted with methanol.

### 4.6. Mass Spectrometry and Data Analysis

The fractions obtained from the chloroform extract, identified as the most effective against cancer cells with limited toxicity towards healthy cells, were analyzed to identify the compounds responsible for the selective antitumor effect. Methanol, acetonitrile and water were LC-MS grade and purchased from VWR, formic acid was LC-MS grade from Sigma Aldrich. The fractions were diluted in a methanol:water mixture (1:1; *v*/*v*) and filtered through a PTFE membrane prior to analysis.

The analysis was performed using an Ultimate 3000 HPLC system coupled with a LTQ Orbitrap XL™ (Thermo Fisher Scientific, Bremen, Germany) mass spectrometer, equipped with a heated electrospray ionization (HESI) source. A total of 19 µL of sample was injected into a Zorbax StableBond Aq column (2.1 × 150 mm, 3.5 µm) from Agilent Technologies, Wilmington, Delaware (USA) and the chromatographic separation was achieved with a mobile phase consisting of solvent A—0.01% formic acid in water—and solvent B—0.01% formic acid in acetonitrile—at a flow rate of 200 µL min^−1^. The gradient elution program was as follows: 0–5 min, 0% B; 5–45 min, gradient to 100% B; 45–60 min, 100% B; 60–61 min, return to 0% B; 61–80 min isocratic equilibration at 0% B. The mass spectrometer operated in full-scan mode with a *m*/*z* range of 80–800, at a resolution of 60,000 at *m*/*z* 400. The source parameters were as follows: capillary temperature, 275 °C; vaporizer temperature, 300 °C; sheath gas flow rate, 35 psi; aux gas flow rate, 5 psi; sweep gas flow rate, 0 psi. Ionization was performed in both negative and positive polarity, using spray voltages of −4 kV and +4 kV, capillary voltages of −43 V and +43 V, and tube lens voltages of −70 V and +70 V, respectively. Each sample was injected in triplicates.

Calibration was performed every 48 h using a mix of standards: caffeine, sodium dodecyl sulfate, sodium taurocholate, MRFA, and Ultramark 1621 (Pierce™ LTQ ESI Positive and Negative Ion Calibration Solutions, Thermo Fisher Scientific, Rockford, IL, USA). To ensure a mass accuracy of <2 ppm, lock masses were used during data acquisition. In positive ionization mode, the lock masses were *m*/*z* 112.9856, 281.2486, and 283.2642, corresponding to formic, stearic, and oleic acids, respectively. In negative ionization mode, the lock masses were *m*/*z* 83.0603 and 158.9640, corresponding to acetonitrile and sodium trifluoroacetate, respectively. The samples were also analyzed by HPLC-HRMS using a data-dependent acquisition method (ddMS^2^). This method simultaneously acquired in full-scan HRMS, MS/MS, and MS^n^ scans of selected precursor ions. During full-scan profiling, the two most intense precursor ions within the selected mass range were fragmented in the linear trap using a normalized collision energy of 35 eV. The most intense fragment ion generated from the most intense precursor was subsequently subjected to MS^3^ fragmentation. Spectra were acquired at a resolution of 15,000.

Due to the unavailability of the LTQ Orbitrap XL™, confirmation analysis of the ionic species at *m*/*z* 297.2428 was performed using an Orbitrap Exploris^TM^ 120 coupled with a Vanquish Flex HPLC system (Thermo Fisher Scientific, Bremen, Germany). The chromatographic method was the same as described above. For MS analysis, the HESI source operated in the negative ionization mode at a spray voltage of −2500 V, with the ion transfer tube at 320 °C and the vaporizer at 300 °C. The mass range was set to *m*/*z* 80–1000 after mass calibration. Mass accuracy was monitored during analysis using EASY-IC™, and data were acquired at a resolution of 120,000. Fragmentation was performed in the HCD (higher-energy collisional dissociation) collision cell with a collision energy of 45 eV.

Data analysis to identify potential bioactive compounds associated with the antitumor activity was performed using Compound Discoverer™ 3.3 software (Figure S1), while Xcalibur™ (Version 4.2.47) was used for data visualization.

The data analysis workflow included the following main steps: RT alignment, unknown compound detection, and compound grouping across all samples. For each detected compound, the elemental composition was first predicted, followed by compound identification by searching against multiple databases: mzCloud™ (using ddMS^2^ data), ChemSpider (using exact mass or elemental formula), and a local database (using mass lists with or without RT). For compounds for which ddMS^2^ data were available, an additional spectral similarity search was performed against mzCloud™. Finally, a differential analysis was performed on the detected compounds (Figure S2).

### 4.7. ROS Production Assay

Kuramochi and MDA-MB-231 cells were seeded in a 96-well plate at a density of 1.5 × 10^3^ cells per well. The cells were treated at predetermined time points (6, 48, and 72 h) with the following compounds: 6-HSA (10 and 20 µM), 6-OSA (15 and 30 µM), and NP-011548 (120 and 240 µM). Doxorubicin was used as positive control at a concentration of 1 µM for Kuramochi cells and 25 µM for MDA-MB-231 cells. For each compound, two concentrations were selected based on preliminary dose–response experiments and compound-specific cytotoxic potency to investigate cellular responses over a biologically active concentration range. The treatment was applied daily, starting from the 72 h time point and scaling down. After 72 h from the initial seeding, ROS production was measured using the ROS-Glo™ H_2_O_2_ Assay.

### 4.8. Caspase Activation

Caspase-3/7 activity was analyzed in Kuramochi and MDA-MB-231 cells seeded at 1500 cells per well in 96-well plates. The following day, cells were treated with the following compounds: cisplatin (10 µM), which served as the positive control, 6-HSA (10 and 20 µM), 6-OSA (15 and 30 µM), and NP-011548 (120 and 240 µM). Untreated cells served as negative control. Caspase-3/7 activity was assessed using the same compound-specific concentrations and exposure times employed in the ROS production assay. For Kuramochi cells, caspase-3/7 activity was assessed at 6 and 48 h post-treatment; for MDA-MB-231 cells, measurements were performed at 6 and 72 h. Caspase-3/7 activity was measured using the Caspase-Glo^®^ 3/7 Assay, and luminescence was analyzed with a Synergy H1 plate reader. Data was normalized to the luminescence of untreated controls.

### 4.9. Transmission Electron Microscopy

Kuramochi and MDA-MB-231 cells were seeded in 10 cm Petri dish at a concentration of 1 × 10^6^ cells per plate. The following day, cells were treated with 100 µM NP-011548 for 24 h. This concentration was selected to obtain detectable ultrastructural alterations within the 24 h exposure period while retaining sufficient cellular integrity for morphological analysis. At the end of the treatment period, cells were detached using trypsin–-EDTA 0.25% and subsequently fixed by immersion in 2.5% glutaraldehyde prepared in 0.1 mol L^−1^ cacodylate buffer (pH 7.2) for approximately 2 h at 4 °C. After fixation, samples were rinsed multiple times in the same buffer and post-fixation was performed in 1% osmium tetroxide, also in 0.1 mol L^−1^ cacodylate buffer, for 2 h at 4 °C. Following this, samples were rinsed in cacodylate buffer, dehydrated through a graded acetone series, and embedded in epoxy resin (Durcupan™ ACM, Sigma-Aldrich, St. Louis, MO, USA) for long-term storage. Ultrathin sections (approximately 75–85 nm thick) were obtained using an Ultracut S ultramicrotome (Leica Microsystems, Vienna, Austria). Sections were stained with Uranyless EM stain, followed by counterstaining with lead citrate. Specimens were examined and imaged using a Talos™ L120C-G2 transmission electron microscope (Thermo Fisher Scientific, Waltham, MA, USA).

## 5. Conclusions

This study identifies (*E*)-6-hydroxyoctadec-4-enoic acid (NP-011548) as a candidate metabolite associated with the selective antitumor activity of *S. lomentaria*. Through a bioassay guided fractionation strategy combined with HRMS, NP-011548 was identified in the active fraction, and its biological activity was subsequently evaluated using the pure compound.

NP-011548 selectively reduced the viability of breast and ovarian cancer cells while exhibiting limited toxicity towards normal fibroblasts. Comparative testing with the structural analogs 6-HSA and 6-OSA further indicated that the C4 double bond and the C6 hydroxy group may contribute to the selective activity of NP-011548. Its cytotoxic effect was not associated with significant caspase-3/7 activation but was accompanied by delayed ROS accumulation and extensive mitochondrial degeneration, lysosomal accumulation, chromatin condensation and plasma membrane alterations, supporting the involvement of a caspase-independent cell death mechanism.

Overall, these findings expand the known biological profile of NP-011548 and support its contribution, at least in part, to the selective antitumor activity of *S. lomentaria*. They also further highlight marine oxylipins as a promising class of bioactive metabolites with potential relevance for anticancer research.

## Figures and Tables

**Table 1:** IC_50_ values (mean ± standard deviation) in µg mL^−1^ for MRC-5, Kuramochi, and MDA-MB-231 cell lines by exposure to extracts and extract fractions and subfractions from *S. lomentaria* and *S. dotyi*. NA = not available.

		IC_50_ (µg mL^−1^)					
		*S. lomentaria*			*S. dotyi*		
		MRC-5	Kuramochi	MDA-MB-231	MRC-5	Kuramochi	MDA-MB-231
Extracts	Milli-Q water	>300	>300	>300	NA	NA	NA
	Methanol	>300	>300	>300	NA	NA	NA
	Acetone	>300	>300	>300	NA	NA	NA
	Chloroform	>300	74 ± 2	79 ± 11	>300	211 ± 16	>300
	Hexane	114 ± 6	43 ± 9	69 ± 12	NA	NA	NA
Primary fractionation from chloroform extract	F1	>300	>300	>300	NA	NA	NA
	F2	>300	141 ± 13	25 ± 5	NA	NA	NA
	F3	69 ± 1	78 ± 4	19 ± 2	NA	NA	NA
	F4	>300	>300	>300	NA	NA	NA
Secondary fractionation from F2 fraction	F2A	131 ± 63	32 ± 4	29 ± 5	38 ± 13	45 ± 3	31 ± 6
	F2B	>300	>300	>300	>300	>300	>300
	F2C	>300	276 ± 88	>300	>300	>300	>300
	F2D	>300	>300	>300	>300	144 ± 39	>300
	F2E	>300	116 ± 24	31 ± 5	>300	>300	>300
	F2F	160 ± 72	>300	>300	>300	>300	264 ± 21
	F2G	>300	208 ± 10	>300	>300	>300	>300

**Table 2:** Ratios and *p*-values calculated for the ionic species *m*/*z* 297.2428.

			Ratio	*p*-Value
Active compound in algae		(F2E-SL)/(F2E-SD)	17.78	3.18 × 10^−14^
Media		(DMEM + F2E-SL)/(DMEM)	96.97	8.81 × 10^−10^
MDA-MB-231	1 h	(F2E-SL)/(Untreated)	10.68	6.11 × 10^−8^
	48 h	(F2E-SL)/(Untreated)	5.45	4.97 × 10^−14^

**Table 3:** IC_50_ values (mean ± standard deviation) for 6-HSA, 6-OSA, and NP-011548 in MRC-5, Kuramochi, and MDA-MB-231 cell lines.

	IC_50_ (µM—(µg mL^−1^))		
COMPOUND	MRC-5	Kuramochi	MDA-MB-231
6-HSA	12.1 ± 1.6	18.7 ± 1.2	3.34 ± 0.45
	(3.6 ± 0.5)	(5.6 ± 0.4)	(1.0 ± 0.1)
6-OSA	79.0 ± 7.7	109 ± 5	17.2 ± 3.5
	(24 ± 2)	(33 ± 1)	(5.1 ± 1.0)
NP-011548	>200	91.9 ± 7.0	91.5 ± 7.1
	(>60)	(27 ± 2)	(27 ± 2)

## Data Availability

The data supporting the findings of this study are available from the corresponding author upon reasonable request.

## Supplementary Materials

The following supporting information can be downloaded at https://www.mdpi.com/article/10.3390/md24100363/s1: Figure S1: Compound Discoverer™ Workflow Tree showing the main steps of the HRMS data processing workflow for compound detection, characterization, and identification; Figure S2: Compound Discoverer™ Statistical Methods section showing the statistical steps applied during data analysis, including those used for differential analysis; Figure S3: Comparison of the fragmentation spectra of the ion at *m/z* 297.2428 detected in the raw file (top) and in the mzCloud™ library reference (bottom); Figure S4: Comparison of the chromatograms (top) and fragmentation spectra (bottom) of NP-011548 (upper) and the unknown compound in F2E-SL (lower); Figure S5: Identification sheet of *Scytosiphon lomentaria*. (A,B) Adult thalli; (C) transverse section of the thallus; (D) presence of ascocysts; (E) higher-magnification view of the ascocysts. Scale bars: (A,B) 5 cm; (C,D) 30 µm; (E) 10 µm; Figure S6: Identification sheet of *Scytosiphon dotyi*. (A,B) Adult thalli in their natural habitat; (C) basal portion of the thallus showing the attachment region and emerging tubular axes; (D) surface view of cortical cells without ascocysts; (E) transverse section of the thallus. Scale bars: (A,B) 5 cm; (C) 1 mm; (D) 10 µm; (E) 75 µm; Figure S7: Extraction and fractionation workflow of *Scytosiphon lomentaria* and *Scytosiphon dotyi*; Table S1: Authors comparison on *Scytosiphon lomentaria* extracts and compounds structurally related to (*E*)-6-hydroxyoctadec-4-enoic acid (NP-011548).

## Abbreviations

The following abbreviations are used in this manuscript:

6-HSA	6-hydroxystearic acid
6-OSA	6-oxostearic acid
7-HSA	7-(*R*)-hydroxystearic acid
7-OSA	7-oxostearic acid
9-HpODE	9-hydroperoxy-octadecadienoic acid
13(*S*)-HPOTrE	13(*S*)-hydroperoxylinolenic acid
dd	Data-dependent
DHA	Docosahexaenoic acid
DMEM	Dulbecco’s modified eagle medium
DMSO	Dimethyl sulfoxide
EDTA	Ethylenediaminetetraacetic acid
EM	Electron microscopy/microscope
EPA	Eicosapentaenoic acid
ER	Endoplasmic reticulum
FBS	Fetal bovine serum
GPC	Gel permeation chromatography
HCD	Higher-energy collisional dissociation
HESI	Heated electrospray ionization
HPLC	High-performance liquid chromatography/chromatograph
HRMS	High-resolution mass spectrometry/spectrometer
IC_50_	Half-maximal inhibitory concentration
*m*/*z*	Mass-to-charge ratio
MEM	Minimum essential medium
MRFA	Met-Arg-Phe-Ala
MS	Mass spectrometry/spectrometer
MS/MS	Tandem mass spectrometry/spectrometer
NP-011548	(*E*)-6-hydroxyoctadec-4-enoic acid
OFAs	Oxygenated fatty acids
PBS	Phosphate-buffered saline
PTFE	Polytetrafluoroethylene
PUFAs	Polyunsaturated fatty acids
ROS	Reactive oxygen species
RT	Retention time
SHFAs	Saturated hydroxy fatty acids
SOFAs	Saturated oxo fatty acids
SPE	Solid phase extraction
STAT3	Signal transducer and activator of transcription 3
TEM	Transmission electron microscopy/microscope
