# Supplementary material for: Identification of an Unsaturated Hydroxy Fatty Acid Antitumor Oxylipin from Brown Alga Scytosiphon lomentaria: (E)-6-Hydroxyoctadec-4-enoic Acid (NP-011548)
Source: Mar Drugs. 2026 Oct 19;24(10):363. doi: 10.3390/md24100363 (PMC13706638; doi:10.3390/md24100363)
Supplement: Supplementary file 1 [file marinedrugs-24-00363-s001.zip › marinedrugs-4580604-supplementary.pdf]

Supplementary Material

Identification of an Unsaturated Hydroxy Fatty Acid Antitumor Oxylipin from Brown Alga *Scytosiphon lomentaria*: (*E*)-6-Hydroxyoctadec-4-enoic Acid (NP-011548)

Matteo Mauceri [1], Abdul-Salam Juhmani [2], Roberta Zangrando [3], Michele Gallo [4], Flavio Rizzolio [4,5], Adriano Sfriso [1], Sarah Pizzini [1,6,7], Marco Roman [1], Andrea Gambaro [1], Carlotta Granchi [8], Tiziano Tuccinardi [8], Giuseppe Forlani [9] and Andrea Augusto Sfriso [9,*]

1 Department of Environmental Sciences, Informatics and Statistics, Ca' Foscari University of Venice, Via Torino, 155, 30172 Venezia, Italy; matteo.mauceri@unive.it (M.M.); sarah.pizzini@cnr.it (S.P.); marco.roman@unive.it (M.R.); gambaro@unive.it (A.G.)

2 Department of Biology and Biotechnology, Faculty of Science, The Hashemite University, Zarqa 13133, Jordan; a.juhmani@hu.edu.jo

3 National Research Council, Institute of Polar Sciences (CNR-ISP), Via Torino, 155, 30172 Venezia, Italy

4 Department of Molecular Sciences and Nanosystems, Ca' Foscari University of Venice, Via Torino, 155, 30172 Venezia, Italy; gallomke@unive.it (M.G.)

5 Pathology Unit, Centro di Riferimento Oncologico di Aviano (CRO), Via F. Gallini, 2, 33081 Aviano, Italy

6 National Research Council, Institute for Marine Biological Resources and Biotechnology (CNR-IRBIM), Largo Fiera della Pesca, 2, 60125 Ancona, Italy

7 Fano Marine Center, The Inter-Institute Center for Research on Marine Biodiversity, Resources and Biotechnologies, Viale Adriatico, 1/N, 61032 Fano, Italy

8 Department of Pharmacy, University of Pisa, Via B. Pisano, 6, 56126 Pisa, Italy; carlotta.granchi@unipi.it (C.G.); tiziano.tuccinardi@unipi.it (T.T.)

9 Department of Life Sciences and Biotechnology, University of Ferrara, Via L. Borsari, 46, 44121 Ferrara, Italy; flg@unife.it

* Correspondence: sfrndr@unife.it

SUPPLEMENTARY MATERIAL

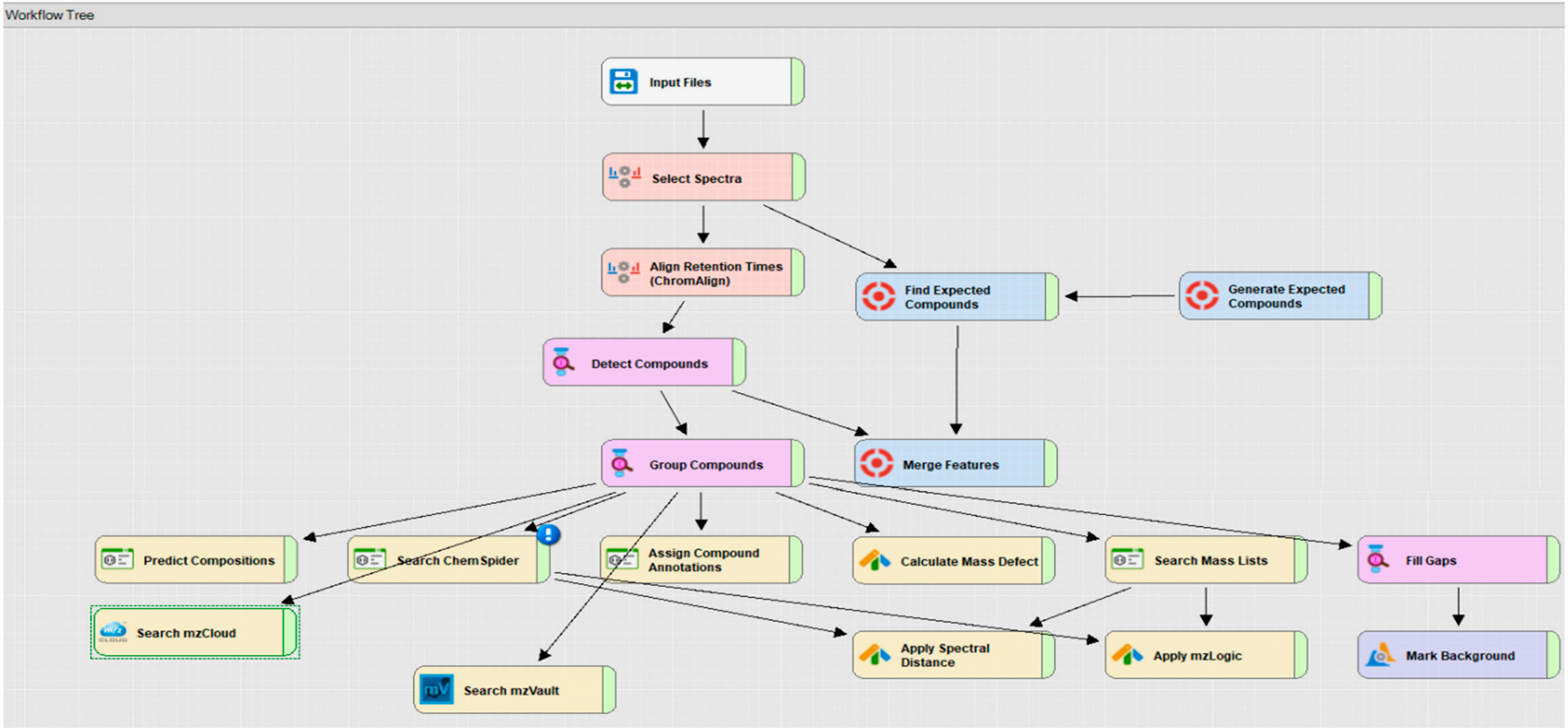

Figure S1. Compound Discoverer™ Workflow Tree showing the main steps of the HRMS data processing workflow for compound detection, characterization, and identification.

Compounds | Compounds per File | Expected Compounds per File | Expected Formulas | Merged Features | Features per File | Expected Features per File | mzVault Results | mzCloud Results | ChemSpid

	Checked	Node Name	Processing Step	Method Description
1		Align Retention Times (ChromAlign)	Retention Time Alignment	Retention time alignment performed by the ChromAlign algorithm: Sadygov et al. (https://doi.org/10.1021/ac060923y)
2		Group Compounds	[Compounds] Apply Peak Rating Filter	Only compounds with an Original Peak Rating greater or equal to 5 in at least 3 samples are kept for further processing.
3		Fill Gaps	Similar Features Search	Features search within a tolerance of 5 ppm
4		Fill Gaps	Centroids Filtering	Filtered centroids with S/N threshold = 3
5		Fill Gaps	Detection	Real detection (for more accurate areas) was performed.
6		Differential Analysis	[Compounds] Input Data	Peak Area
7		Differential Analysis	[Compounds] Data Transformation	log-10 areas for p-value estimation
8		Differential Analysis	[Compounds] Statistical Test	The p-value of per group ratio calculated by a one-way ANOVA model with Tukey as post-hoc test.
9		Differential Analysis	[Compounds] p-value Correction	p-value adjusted using Benjamini-Hochberg correction for the false-discovery rate.

Figure S2. Compound Discoverer™ Statistical Methods section showing the statistical steps applied during data analysis, including those used for differential analysis.

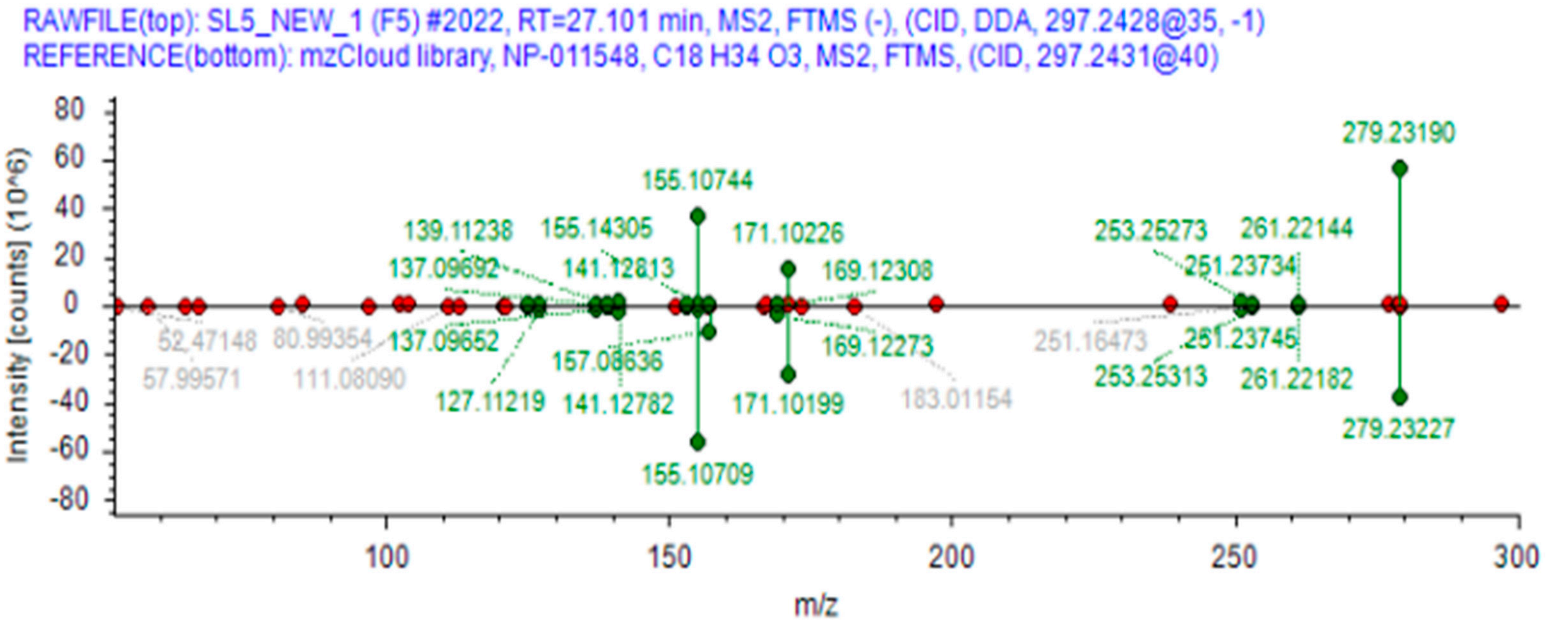

Figure S3: Comparison of the fragmentation spectra of the ion at *m/z* 297.2428 detected in the raw file (top) and in the mzCloud™ library reference (bottom).

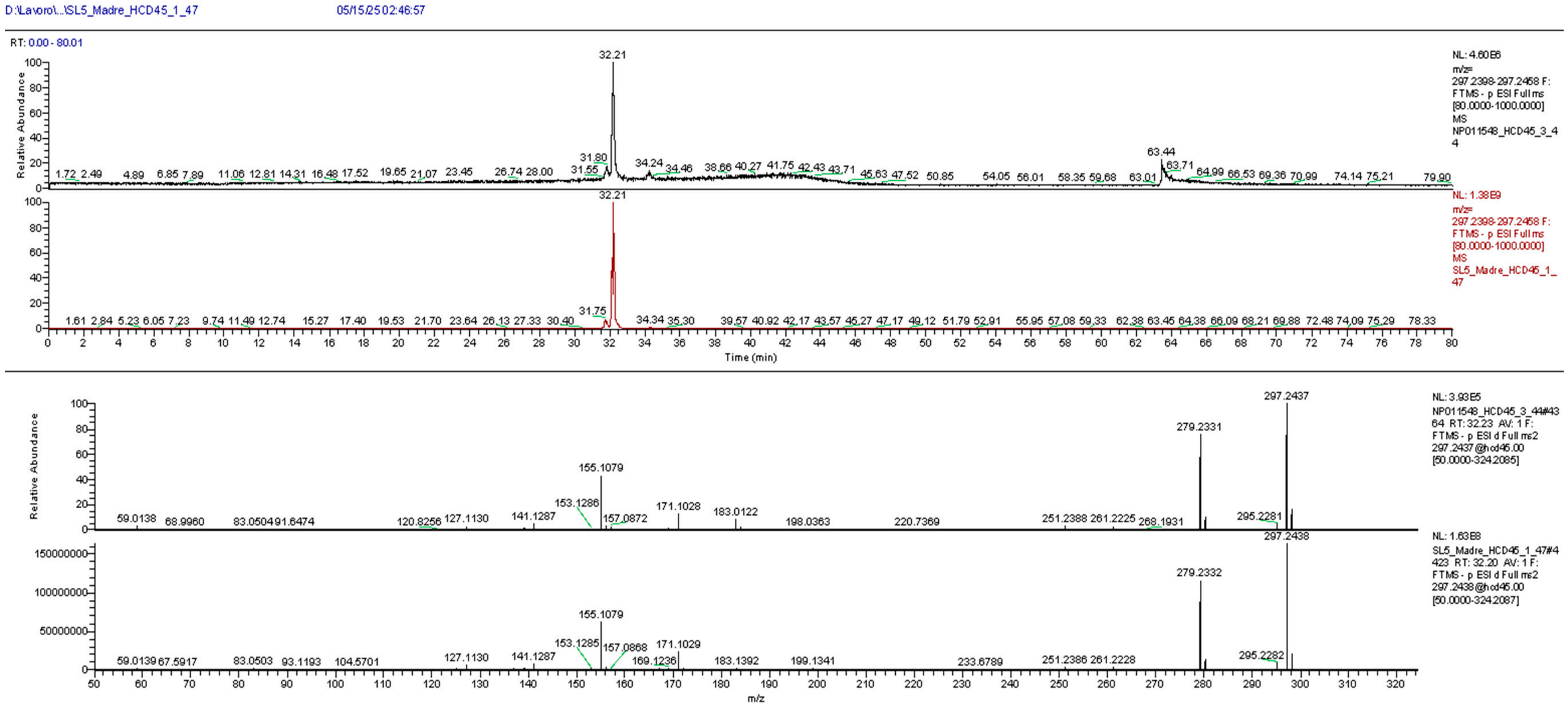

Figure S4: Comparison of the chromatograms (top) and fragmentation spectra (bottom) of NP−011548 (upper) and the unknown compound in F2E−SL (lower).

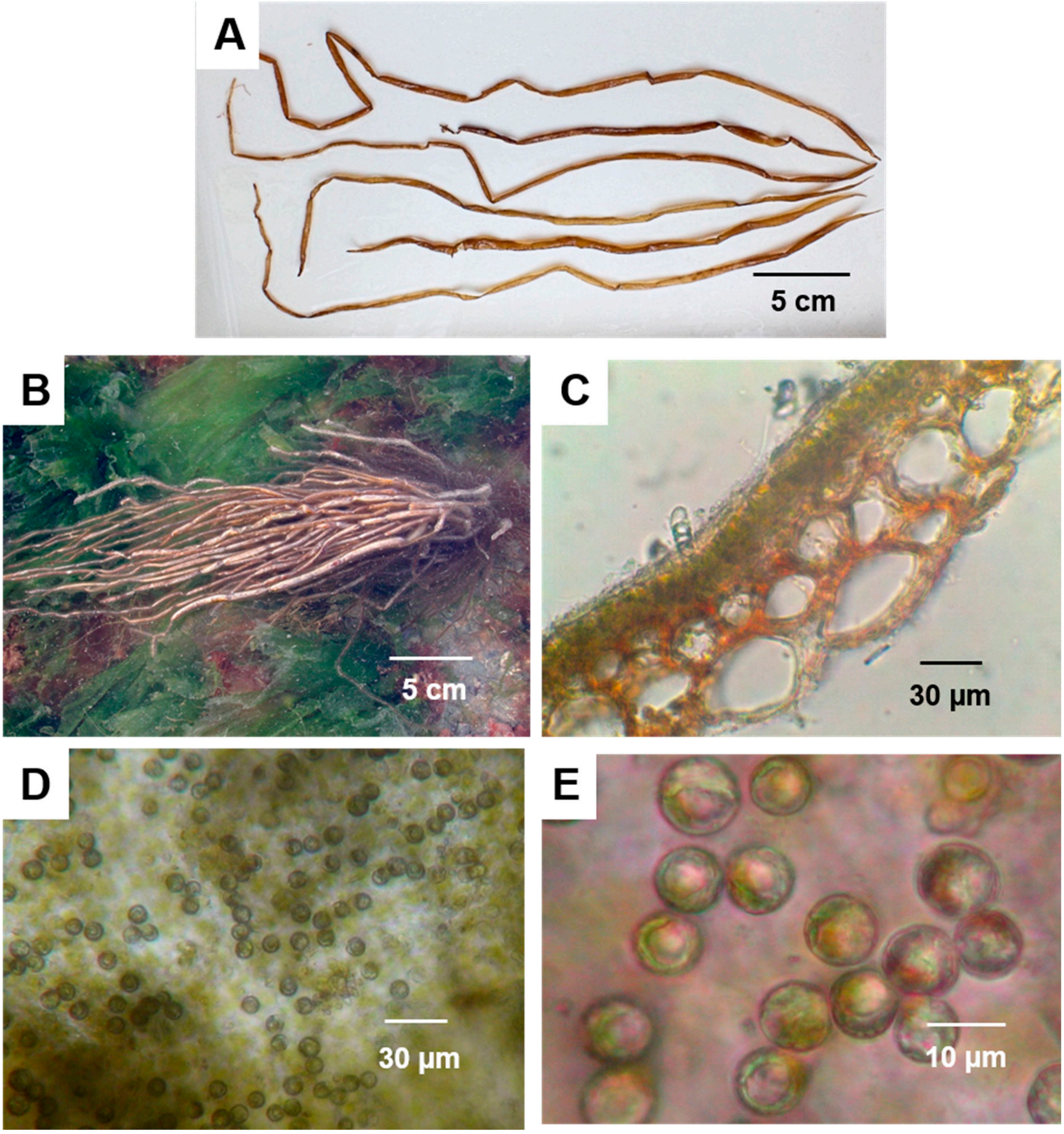

Figure S5. Identification sheet of *Scytosiphon lomentaria*. (A,B) Adult thalli; (C) transverse section of the thallus; (D) presence of ascocysts; (E) higher–magnification view of the ascocysts. Scale bars: (A,B) 5 cm; (C,D) 30 μm; (E) 10 μm.

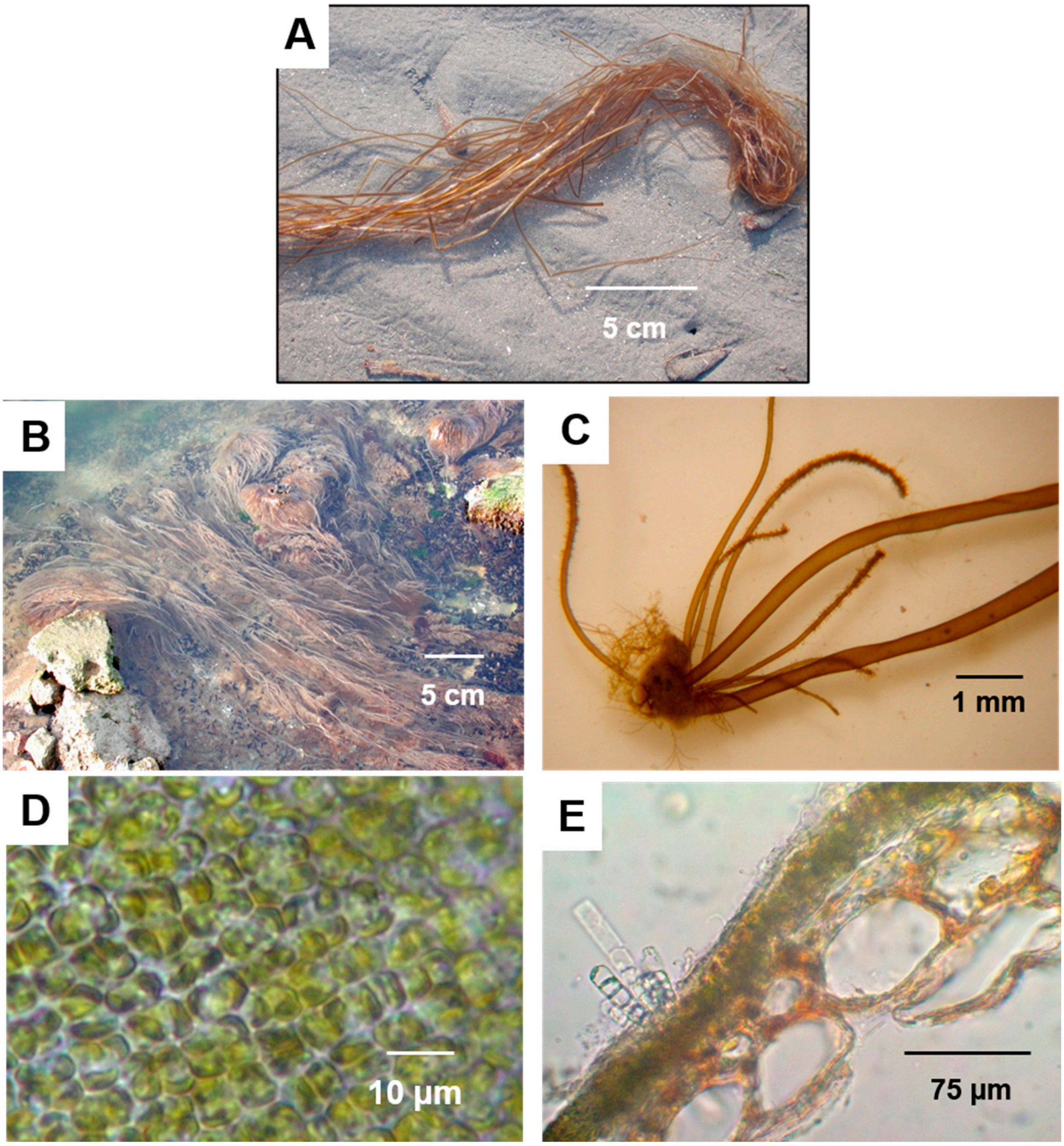

Figure S6: Identification sheet of *Scytosiphon dotyi*. (A,B) Adult thalli in their natural habitat; (C) basal portion of the thallus showing the attachment region and emerging tubular axes; (D) surface view of cortical cells without ascocysts; (E) transverse section of the thallus. Scale bars: (A,B) 5 cm; (C) 1 mm; (D) 10 µm; (E) 75 µm.

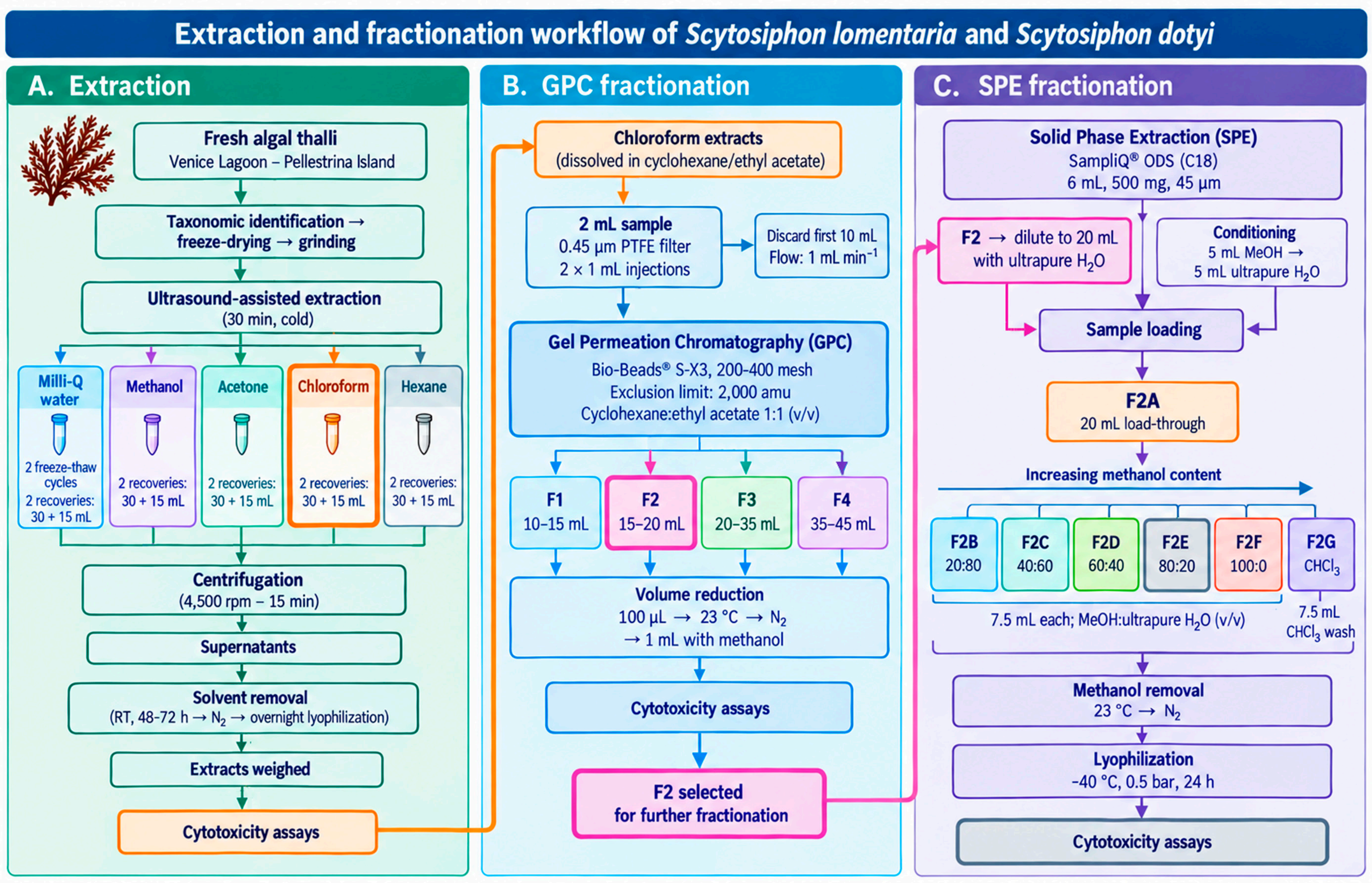

Figure S7. Extraction and fractionation workflow of *Scytosiphon lomentaria* and *Scytosiphon dotyi*.

Table S1. Authors comparison on *S. lomentaria* extracts and compounds structurally related to (E)−6−hydroxyoctadec−4−enoic acid (NP−011548).

Authors	Matrix	Vitality tests	Cell lines tested	Effects
**Xu et al. (2004)**	Ethanol algae extract	MTT	Human oral epidermoid carcinoma KB; human colon cancer line HT−29; human normal cells NIH−3T3	Selective toxicity against KB and HT−29
**Noda et al. (1990)**	Autoclaved alga	Mice implanted with Ehrlich carcinoma (solid) − oral administration daily (1.6g day^{-1} kg^{-1}) for 30 days of raw alga − tumoral solid mass weight − growth inhibition	Ehrlich carcinoma (solid)	significant inhibition rate = 69.8%
**Kim et al. (2004)**	EtOAc algal extract	MTT; apoptosis; DNA fragmentation; Western blot: c−Myc, Bcl−2, Bax, caspase−3, PARP	HL−60 promyelocytic leukemia cells; SNU−16 stomach cancer cells; KG−1myelogenous leukemia cells; MCF−7 breast cancer cells; A549 lung cancer cells; A498 kidney cancer cells; SKOV3 ovary cancer cells; HEL−299 fibroblasts cells	Marked inhibition on leukemia cells (41−43%), lower effect on others; apoptosis induction; decrease in c−Myc after 24 hours dose dependent; down−regulation Bcl−2 and activation of caspase; selective effect − no effects on HEL−299 fibroblasts cells; activation/increase of the active form of caspase−3
**Batsika et al. (2021)**	PURE COMPOUNDS − Saturated oxo fatty acids (OSAs) − ketone from 4−10	MTT; Western blot: cyclin D1, cyclin E1, EGFR, ERBB2 (Her2), PI3K, p53, mTOR, PTEN, AKT1, c−Myc, STAT 3	Epithelial lung carcinoma A549; colorectal adenocarcinoma Caco−2; astrocytoma SF268; normal human umbilical vein endothelial cells HUVEC	5−6−7−8−OSA inhibition growth higher than 50%. Effect from 10 to 75μM; 6−7 OSA most potent IC50=26−28μM; no effect on normal cells up to 75μM; STAT3 and c−Myc reduced in presence of 6−7−OSA; compounds naturally present in human cells.
**Kokotou et al. (2020)**	PURE COMPOUNDS − Saturated hydroxy fatty acids (HSAs) hydroxy 7−9	MTT; Immunofluorescence (phospho−H3, activated caspase−3); RT−qPCR + Western blot (STAT3 pathway)	A549 epithelial lung carcinoma; Caco−2 colorectal adenocarcinoma; SF268 astrocytoma; U87−MG glioblastoma; INS−1 β−cells.	7−HSA found in bovine milk; 7− and 9−HSA/HPA most active; 7(R)−HSA most potent (IC_{50} = 38 μM); no effect of stearic acid; cell−cycle arrest without apoptosis; decreased phospho−histone H3; no increase in activated caspase−3; downregulation of STAT3 mRNA and total/phosphorylated STAT3 protein; no significant changes in cyclin D1, cyclin E1, EGFR, PI3K, p53, mTOR, c−Myc or RET

References

Batsika, C.S.; Mantzourani, C.; Gkikas, D.; Kokotou, M.G.; Mountanea, O.G.; Kokotos, C.G.; Politis, P.K.; Kokotos, G. Saturated oxo fatty acids (SOFAs): A previously unrecognized class of endogenous bioactive lipids exhibiting a cell growth inhibitory activity. *J. Med. Chem.* **2021**, *64*, 5654–5666, https://doi.org/10.1021/acs.jmedchem.0c02058.

Kim, S.C.; Park, S.Y.; Hyoun, J.H.; Cho, H.; Kang, J.H.; Lee, Y.K.; Park, D.B.; Yoo, E.S.; Kang, H.K. The cytotoxicity of *Scytosiphon lomentaria* against HL−60 promyelocytic leukemia cells. *Cancer Biother. Radiopharm.* **2004**, *19*, 641–648, https://doi.org/10.1089/cbr.2004.19.641.

Kokotou, M.G.; Kokotos, A.C.; Gkikas, D.; Mountanea, O.G.; Mantzourani, C.; Almutairi, A.; Lei, X.; Ramanadham, S.; Politis, P.K.; Kokotos, G. Saturated hydroxy fatty acids exhibit a cell growth inhibitory activity and suppress the cytokine−induced β−cell apoptosis. *J. Med. Chem.* **2020**, *63*, 12666–12681, https://doi.org/10.1021/acs.jmedchem.0c00844.

Noda, H.; Amano, H.; Arashima, K.; Nisizawa, K. Antitumor activity of marine algae. *Hydrobiologia* **1990**, *204*, 577–584, https://doi.org/10.1007/BF00040290.

Xu, N.; Fan, X.; Yan, X.; Tseng, C.K. Screening marine algae from China for their antitumor activities. *J. Appl. Phycol.* **2004**, *16*, 451–456, https://doi.org/10.1007/s10811−004−5508−x.
